# Industrial Product Quality Analysis Based on Online Machine Learning

**DOI:** 10.3390/s23198167

**Published:** 2023-09-29

**Authors:** Yiming Yin, Ming Wan, Panfeng Xu, Rui Zhang, Yang Liu, Yan Song

**Affiliations:** 1 School of Physics, Liaoning University, Shenyang 110036, China; yinym6517@mails.jlu.edu.cn (Y.Y.); xupanfeng@lnu.edu.cn (P.X.); 15940256305@163.com (R.Z.); yangliu@lnu.edu.cn (Y.L.); 2 College of Information, Liaoning University, Shenyang 110036, China; wanming@lnu.edu.cn

**Keywords:** industrial product quality analysis, identity parsing, online machine learning

## Abstract

During industrial production activities, industrial products serve as critical resources whose performance is subject to various external factors and usage conditions. To ensure uninterrupted production processes and to guarantee the safety of the production personnel, a real-time analysis of the industrial product quality and subsequent decision making are essential. Conventional detection methods have inherent limitations in meeting the real-time demands of processing large volumes of data and achieving high response speeds. For instance, the regular inspection and maintenance of cars can be time-consuming and labor-intensive if performed manually. Furthermore, monitoring the damage situation of bearings in real time through a manual inspection may lead to delays and may hinder production efficiency. Therefore, this paper presents online machine-learning-based methods to address these two practical problems and simulates them on various datasets to meet the requirements of efficiency and speed. Prior to being fed into the network for training, the data undergo identity parsing to transform them into easily identifiable streaming data. The training process demonstrates that online machine learning ensures timely model updates as small batches of data are sent to the network. The test results indicate that the online learning method exhibits highly stable and effective performance, optimizing the training process.

## 1. Introduction

Industrial products purchased for processing or business operations play a crucial role in both daily life and production. The quality of these industrial products directly impacts the efficiency and overall quality of the production process. Therefore, even a slight deviation in their condition cannot only reduce their lifespan but can also pose potential risks to the safety of the production personnel, leading to irreparable losses. Moreover, any product malfunction indicates underlying issues with both the manufacturing processes and quality control in the industry, which can result in significant social problems and adverse effects. Hence, the timely evaluation of their quality is crucial and imperative.

Industrial products and their application environments require tailored solutions to meet their specific needs. As a pivotal industrial product and an indispensable vehicle in contemporary society, the automobile serves multifarious functions, such as facilitating transportation, reducing travel time, and ensuring travel quality. There are numerous indicators that can be assessed for any vehicle, including the interconnectivity of its body-frame components, the performance of its associated locomotive mechanism, and the proper functioning of its electrical components [1]. These factors directly impact the vehicle’s price assessment, classification, and future usability. In practical production and application processes, the indicators that can be taken into consideration are more intricate and diverse. Additionally, given the extensive number of vehicles involved, meeting the real-time requirements for both production and safety inspections through conventional methods poses significant challenges. The literature [2] presents a systematic model for evaluating internet cars where user evaluations are analyzed using feature extraction and LSTM, while the overall training process incorporates DCGAN. This approach enables the utilization of internet-based model evaluations as a reference for car buyers and facilitates product optimization for automotive companies. The literature [3] presents a framework that enables the assessment of autonomous vehicles’ condition and their surrounding environment in real-world scenarios. The proposed framework encompasses a range of software and hardware facilities along with a complete set of systems comprising the environmental perception layer, behavioral decision-making layer, and motion planning layer. The effectiveness of this framework has been validated through its application to two natural driving datasets encompassing both typical driving situations and high-risk scenarios. The literature [4] proposes an accelerated evaluation concept through the utilization of piecewise mixture distribution models. The test results demonstrate that this approach reduces the evaluation time and significantly enhances accuracy and efficiency. Furthermore, in addition to the overall assessment of a vehicle’s condition, there exist various methodologies for evaluating the specific indicators pertaining to different types of vehicles. In the literature [5], two approaches, namely, subjective and objective methods, have been proposed to analyze their correlation and to evaluate the comfort level of a vehicle based on diverse feedback from both drivers and vehicles. The literature [6] has developed an intelligent connected vehicle (ICV) information collection system platform for monitoring the driving state of intelligent vehicles. It utilizes an immune algorithm model to analyze and evaluate real-time driving data. The experimental results demonstrate that the evaluation outcomes align with actual states, indicating the feasibility and effectiveness of this approach. In the realm of autonomous vehicles, the literature [7] proposes a novel evaluation approach that integrates chassis-domain modeling with spatiotemporal signal analysis during driving to establish a connection to the overall vehicle performance, encompassing safety and comfort. For long combination vehicles (LCVs), the literature [8] proposes a novel evaluation method for dynamic stability based on simulation experiments. This study aims to investigate the underlying factors contributing to the divergent outcomes observed across various evaluation methods.

The bearing is a crucial component of modern industrial equipment, serving the primary function of providing support to mechanical rotating bodies, reducing friction coefficients during movement, and ensuring rotational accuracy [9,10]. The precision, performance, lifespan, and reliability of bearings are all vital in the production process. In industry settings, bearings are considered high-precision products that require comprehensive support from various disciplines, such as mathematics and physics alongside material science, heat treatment technology, precision machining and measurement technology, and numerical control technology, with effective numerical methods supported by powerful computer technologies to serve them effectively. Therefore, the fault diagnosis of bearings plays a pivotal role in assessing the manufacturing quality, while studying bearing fault diagnosis techniques remains critical. The current methodology for diagnosing bearing faults primarily relies on the utilization of the CWRU dataset. The literature [11] proposes a WDCNN algorithm that utilizes the original time signal from the CWRU dataset as an input and employs a large-size convolution kernel in the first convolution layer to effectively suppress high-frequency noise. Subsequently, smaller convolution kernels are utilized for multilayer nonlinear mapping, ultimately achieving a nearly 100% classification accuracy. In the field of bearing defect diagnosis and spectrogram creation, a novel approach is introduced in the literature [12] to effectively reduce input data dimensionality and to optimize the model structure by downsampling vibration sensor signals. This specific method employs a lightweight CNN model with fixed feature map dimensions, resulting in a high classification accuracy with low-dimensional input data. In the literature [13], a TCNN network, also known as ResNet-50, is introduced with 51 convolutional layers. The research proposes a method to convert the time-domain signal of the CWRU dataset into the RGB image format for the network input, resulting in an impressive testing accuracy rate of 99.99%. The literature [14] proposes an enhanced neural network model based on AlexNet, which effectively addresses the overfitting issue commonly encountered in deep neural networks through the utilization of transfer learning. An impressive classification accuracy of 99.7% is achieved on the CWRU dataset using their method. The literature [15] addresses the challenges of irreversible demagnetization faults and bearing faults by employing the transfer learning method and VGG-16 network, utilizing the pretrained VGG-16 parameters from ImageNet. As a result, it achieves an impressive accuracy rate of 96.65% in fault classification. The literature [16] introduces a convolutional neural network called TICNN, which integrates training interference to enhance bearing fault detection. The results demonstrate the algorithm’s robust adaptability to diverse environments and its effective performance in noisy settings without prior noise reduction.

Considering the aforementioned factors, numerous contemporary algorithms demonstrate an almost perfect recognition accuracy of nearly 100% on both the car evaluation dataset employed in vehicle quality detection and the CWRU dataset utilized in bearing fault classification. Therefore, it is imperative to shift our focus towards the practical implementation of these methods rather than solely pursuing the improvement of the algorithm [17,18]. Consequently, we have resolved to evaluate the industrial product quality through online machine learning, which can efficiently detect real-time issues while being better suited for actual environments. The primary contributions of this paper are as follows:

- The quality issues of industrial products, such as cars and bearings, are effectively addressed through the implementation of an online machine learning method and are verified on relevant datasets;
- After undergoing data preprocessing and identification analysis, the pertinent datasets are inputted into the network to enhance the comprehensiveness of the extracted features and to improve the accuracy of the prediction results;
- The initial WDCNN model is adapted to better suit the online machine learning requirements;
- Python’s deep river package is utilized to train and predict neural networks in an online learning manner.

**Figure 1:**
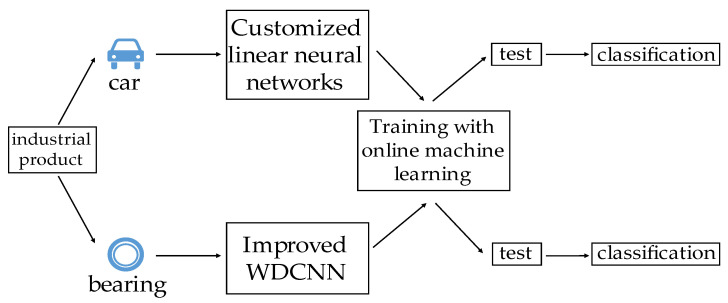
The full-text flow chart.

This paper is organized as follows: Section 2 provides an introduction to the method of online machine learning, specifically focusing on the FTRL algorithm, the deep river library, the description of the dataset, and the proposed solutions; Section 3 provides the comprehensive experimental data and comparisons with various verified methods; Section 4 analyzes and discusses the experimental results as well as the virtues and weakness of our method; and Section 5 concludes our work. The full-text flow chart is shown in Figure 1.

## 2. Methods of Online Machine Learning

### 2.1. Online Machine Learning

The concept of online machine learning does not refer to a specific model but rather signifies a method for training models. The process of online learning diverges from offline learning or batch learning. In traditional training methods, the model’s update cycle is relatively lengthy once it goes online. Additionally, the model remains static and lacks interaction under real-time situations. If a prediction is erroneous, correction can only take place during the subsequent update iteration. However, in online learning, training samples are sequentially inputted into an algorithm, and each sample is utilized only once and is used separately for the training and testing processes, eliminating the necessity to accumulate all the training samples for simultaneous learning [19,20,21,22,23,24]. More specifically, the method of online machine learning initially focused on addressing logistic regression problems. In the context of logistic regression, overfitting can be prevented by minimizing the structural risk, which is so-called regularization, as well as optimizing the loss function. The logistic regression problem at hand can be expressed in two distinct forms. One is the unconstrained optimization form of soft regularization formulation, which is represented by the following formula:

(1) $$\hat{W}=arg\;\underset{W}{\min}\sum_{i=1}^{n}L\left(W,z_{i}\right)+g{\left\|W\right\|}_{1}$$

The other is the convex optimization problem with constraints, whose formula is as follows:

(2) $$h\left(X\right)=-\int_{a}^{b}f\left(x\right)\log\left(f\left(x\right)\right)dx$$

**Figure 2:**
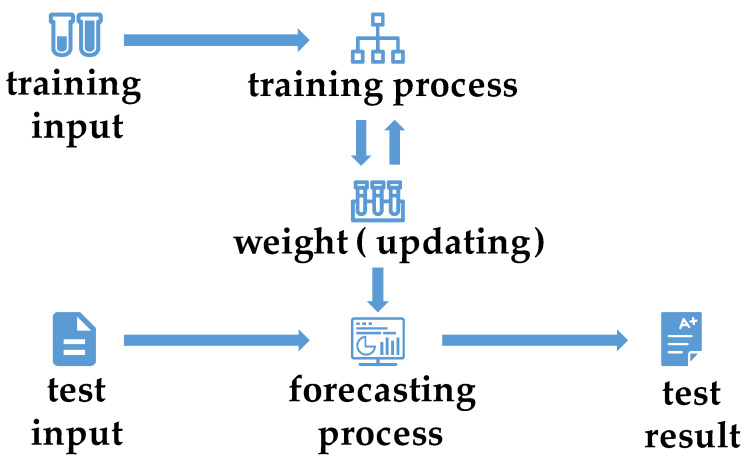
Flowchart of online machine learning.

In Formulas (1) and (2), $L\left(W,z_{i}\right)$ represents the loss function, and g represents the gradient. By adjusting the loss value and the change in the gradient in a timely manner, the learning model can be promptly updated upon receiving new data during the training process. This fundamental distinction distinguishes online learning from offline learning. The advantage of online machine learning lies in its ability to automatically update the model with feedback data, eliminating the need for manual adjustments after training. By incorporating real-time feedback data, online learning can dynamically adjust the model and can enhance prediction accuracy. Figure 2 illustrates the flow chart of online machine learning.

The characteristics of online learning, as illustrated in Figure 2, encompass the acquisition of data from streaming sources, the capability to train new models on minibatches or even single samples, and the ability to generate features in real time. Therefore, if a training process satisfies all of these aforementioned conditions, it can be considered an instance of online machine learning.

### 2.2. FTRL Optimizer

The field of online machine learning primarily focuses on logistic regression problems, with solutions predominantly relying on online gradient descent (OGD) [25,26,27,28] and stochastic gradient descent (SGD), or the gradient descent method [29,30,31]. The typical weight-updating formula of the gradient descent method is shown below:

(3) $$W_{t+1}=\prod_{C}\left(W_{t}-\alpha_{t}(g_{t}+\xi_{t})\right)$$

In Formula (3), $g_{t}$ represents the subgradient of the loss function. To manage the model complexity, the L1 or L2 norm is commonly incorporated into the loss function for regularization purposes. This approach effectively prevents overfitting by penalizing excessive parameters. The L1 norm represents the sum of the absolute values in a vector, also known as Lasso regularization, whereas the L2 norm represents the sum of the squares in a vector, also known as Ridge regression. The weight-updating formula of L1 regularization can be expressed as follows:

(4) $$W_{t+1}=W_{t}-\eta_{t}G_{t}-\eta_{t}\lambda \;sgn\left(W_{t}\right)$$

In Formula (4), $W_{t}$ denotes the weight of the training step t, $\eta_{t}$ represents the learning rate, $G_{t}$ represents the gradient, λ represents the regularization parameter, and $sgn\left(W_{t}\right)$ represents the derived function of $\left|W_{t}\right|$. The mixed regularization formula for the gradient descent method is presented below:

(5) $$\Psi \left(x\right)=I_{C}\left(x\right)+\psi (x)$$

The term $\xi_{t}$ in Formula (3) represents the gradient of $\Psi \left(x\right)$ as defined in Formula (5), with the constraint space C serving as the projection set. The fundamental concept underlying this approach is to perform gradient descent on the loss function for the individual data as described in Formulas (1) and (2). However, due to the suboptimal directions taken by each step of gradient descent and the challenges associated with achieving truly sparse solutions through online gradient descent, there are inherent limitations to its effectiveness. The problem was effectively addressed by Google through the implementation of the follow the regularized leader (FTRL) algorithm, which seamlessly translates theoretical research into practical engineering. The FTRL algorithm is an online learning optimization technique that is particularly well suited for processing vast amounts of data with numerous sparse features. It offers convenience, practicality, and excellent predictive performance. This algorithm exhibits exceptional performance in convex optimization problems featuring nonsmooth regularization terms.

The FTRL algorithm integrates the forward–backward splitting method (FOBOS), an enhanced version of online gradient descent (OGD) [32], and regularized dual averaging (RDA) to effectively harness the characteristics and advantages of both FOBOS and RDA [33]. FOBOS decomposes each datum’s iterative process into an experiential loss gradient descent iteration and an optimization problem, while RDA is a nongradient descent method that aggregates the gradients of each sample to facilitate a smoother gradient-updating process. In contrast to SGD, which solely estimates and descends gradients for one observed sample at a time, RDA can overcome the loss-function oscillation problem of SGD, enhance the sparsity, and prevent the truncation of some dimensions due to insufficient training. Both methods aim to obtain sparser solutions.

The fundamental principle of FOBOS lies in both the historical outcomes and the pursuit of optimal results at this stage. The specific formulas for weight updating are as follows:

(6) $$W_{t+\frac{1}{2}}=W_{t}-\eta_{t}G_{t}$$

(7) $$W_{t}=arg\;\underset{W}{\min}\{\frac{1}{2}{\left\|W-W_{t+\frac{1}{2}}\right\|}^{2}+\eta_{t+\frac{1}{2}}\;\Psi \left(W\right)\}$$

Formula (6) represents standard random gradient descent, whereas Formula (7) denotes the fine tuning of the results based on the current conditions. The following state formula can be derived by combining Formulas (6) and (7):

(8) $$W_{t+1,i}=sgn\left(W_{t,i}-\eta_{t}g_{t,i}\right)max\{0,\left|W_{t,i}-\eta_{t}g_{t,i}\right|-\eta_{t+\frac{1}{2}}\lambda \}$$

Formula (8) implies that the gradient generated by a sample may not be sufficient to elicit a significant weight update in the corresponding dimension. However, RDA accounts for the cumulative change in the gradient and relinquishes the idea of gradient descent. The specific iterative formula is as follows:

(9) $$W_{t}=arg\;\underset{W}{\min}\{\frac{1}{t}\sum_{r=1}^{t}\left\langle G_{r},\left.W\right\rangle \right.+\lambda {\left\|W\right\|}_{1}+\frac{\gamma }{2\sqrt{t}}{\left\|W\right\|}_{2}^{2}\}$$

In Formula (9), $\left\langle G_{r},\left.W\right\rangle \right.$ represents the average integral value of the gradient $G_{r}$ over W, $\lambda {\left\|W\right\|}_{1}$ denotes the L1 regularization term, and $\frac{\gamma }{2\sqrt{t}}$ is a nonnegative nondecreasing sequence. Specifically, the principle of RDA is to replace the original gradient descent method in the solution process with the process of updating the closed-form solution. To prevent overfitting, RDA employs two main strategies. Firstly, RDA introduces a regularization term that constrains the feature weights and reduces their correlation, thereby achieving regularization. Secondly, during the optimization process of the loss function in RDA, if the absolute value of the cumulative average gradient in a certain dimension falls below a threshold value, then the weight for that dimension is reset. The above formulas yield the following result:

(10) $$W_{t+1,i}=\left\{\begin{array}{c} 0,\left|\bar{g_{t,i}}\right|<\lambda \\ -\frac{\sqrt{t}}{\gamma }\left(\bar{g_{t,i}}-\lambda sgn\left(\bar{g_{t,i}}\right)\right),otherwise \end{array}\right.$$

Formula (10) reveals that the fundamental principle of RDA is to assign a weight of zero when the absolute value of the cumulative gradient average generated by a dimension falls below λ. The distinction between FOBOS and RDA lies in their computation methods, with the former calculating the current gradient and L1 regularization terms, while the latter employs cumulative gradients and L1 regularization terms; furthermore, there are variations in the restrictions imposed on W. Despite achieving increased sparsity, RDA exhibits a slight decrease in accuracy. Therefore, as its name suggests, the FTRL algorithm incorporates and balances the advantages of the previous algorithms while considering both the accuracy of FOBOS and the sparsity of RDA [34,35]. Its feature-weight-updating formula can be expressed as follows:

(11) $$W_{t+1}=arg\;\underset{W}{\min}(g_{1:t}W+\frac{1}{2}\sum_{s=1}^{t}\sigma_{t}{\left\|W-W_{s}\right\|}_{2}^{2}+\lambda_{1}{\left\|W\right\|}_{1})$$

The term $g_{1:t}W$ in Formula (11) represents the cumulative sum of the gradients, indicating the direction of the decreasing loss function; $\frac{1}{2}\sum_{s=1}^{t}\sigma_{t}{\left\|W-W_{s}\right\|}_{2}^{2}$ ensures that the predicted results do not deviate significantly from the existing ones; and $\lambda_{1}{\left\|W\right\|}_{1}$ is a regularization term employed to generate sparse solutions. It can be observed that the FTRL algorithm comprehensively considers the impact of both the cumulative weight and the gradient, leading to enhanced accuracy and sparsity.

### 2.3. Deep River

River [36] is a python library designed for constructing online machine learning models that operate on data flows, which consist of sequences of individual elements containing attribute characteristics and parameter information. The objective of river is to serve as a versatile tool for machine learning capable of performing tasks such as classification, regression, anomaly detection, and time-series prediction as well as other streaming-based tasks. In essence, river leverages machine learning models to accomplish tasks through online learning. Additionally, many batch machine learning algorithms incorporate online learning components.

Deep river, an improved version of river and river’s compatibility wrapper for deep learning, is also a python library for online deep learning. The objective of deep river is to facilitate online machine learning for neural networks by integrating the river API with the capabilities of designing neural networks based on PyTorch library. Deep river empowers users to construct diverse types of neural networks in accordance with the design principles of rivers. Currently, deep river can perform most tasks that are achievable by neural networks, such as classification and regression.

Similar to the principle of river, deep river is capable of online data reading, processing one or small batches of samples at a time. Throughout each step of the training process, timely updates can be made to the model parameters while simultaneously extracting features in real time and splicing them into samples. As a result, deep river is fully equipped for online neural network training and prediction.

### 2.4. Datasets

#### 2.4.1. Car Evaluation Dataset

The car evaluation dataset [37] was initially proposed to demonstrate an expert system for decision making, and it was subsequently utilized in the testing of the constructive induction and structure discovery methods. It assesses the condition of each vehicle individually based on six indicators, namely, the buying price, price of maintenance, number of doors, capacity in terms of the persons to be carried, the size of the luggage boot, and estimated safety of the car. The dataset contains a total of 1728 pieces of data. The automobile evaluation results and each indicator encompass multiple states as presented in Table 1.

#### 2.4.2. Case Western Reserve University Dataset

The CWRU dataset is a collection of vibration signals from Western Reserve University that was specifically designed for research on bearing fault diagnosis and life prediction. The provided parameters include the number of samples for each type of fault, the length of each sample, the associated load size, and the degree of fault presentation.

**Figure 3:**
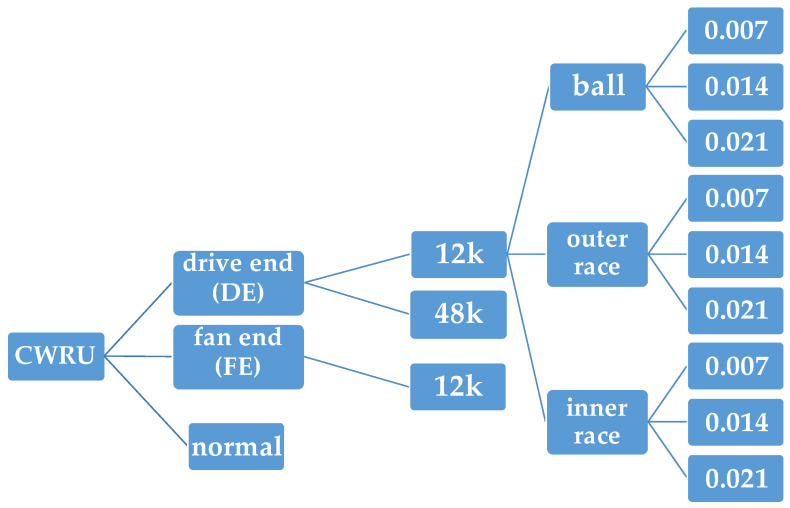
Bearing fault classifications for the CWRU dataset.

The experimental platform comprises a motor, a torque sensor, a power tester, and electronic controllers. The rotating shaft of the motor is supported by the bearings under test. The drive-end bearing is SKF6205, with sampling frequencies of 12 KHz and 48 KHz, while the fan-end bearing is SKF6203, with a sampling frequency of 12 KHz. After measurement, the experimental data are processed into mat files containing the vibration data for both the fan- and drive-end bearings as well as the motor speed information. The final sample data include 4 normal samples, 77 outer-race damage samples, 40 inner-race damage samples, and 40 rolling-body damage samples. Each sample mat file contains drive-end accelerometer data, fan-end accelerometer data, base accelerometer data, time-series data, and revolutions-per-minute values. Among them, a one-dimensional time-series signal was mainly utilized in this paper. The classification of bearing faults includes three types: the inner raceway, outer raceway, and rolling element. Each type is further divided into different cases based on the fault diameters (7 mils, 14 mils, and 21 mils). Overall, nine distinct fault types exist in addition to the normal conditions, resulting in ten possible classifications for bearing faults. The specific classifications of bearing faults are shown in Figure 3.

To enhance the accuracy and representativeness of the research content, this paper exclusively utilized the experimental data obtained from SKF6205, a deep-groove ball bearing with a sampling frequency of 12 kHz. A total of 10,000 pieces of data was selected, comprising 36 mat files, representing the inner raceway, outer raceway, and rolling element, along with 4 normal files. The nine types of bearing damage and one normal type are denoted by the letters A to J for the sake of convenience in the description. Table 2 presents the categories and corresponding quantities of the dataset that was used as well as the corresponding letter representation for each type.

### 2.5. Solutions to Different Problems in the Quality of Industrial Products

**Figure 4:**
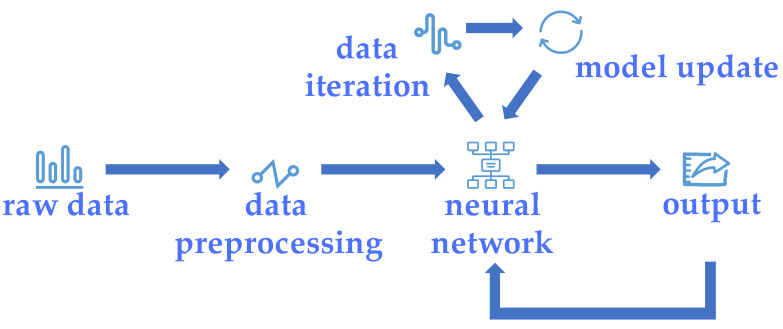
The process of online learning in this paper.

The car evaluation dataset and CWRU dataset necessitate distinct approaches due to the disparities in their data format, content, and quantity. This entails distinct schemes for data preprocessing and the neural network structure. However, both problems exhibit similarities in their training and testing processes, as they both utilize the online learning mode based on deep river and the FTRL optimizer. Figure 4 illustrates the specific learning process.

The raw data of the dataset in Figure 4 were subjected to manual data preprocessing or river data preprocessing, enabling their conversion into a format compatible with neural networks. Subsequently, during the training process, each set of data underwent iteration and updating using river’s metric library. Following the model updates, the evaluation indicators were also updated, and the current score value along with the classification were outputted. The current output was utilized as a reference for the training of the next set of data.

#### 2.5.1. Solution to Car Evaluation

Due to the limited amount of data in the car evaluation dataset and its simple structure, it may be more appropriate to employ a simpler neural network and to reduce the number of training samples fed into the network during each iteration. This approach can not only accelerate the training speed but can also enhance the real-world detection performance.

The overall neural network comprises four linear layers, where the first two incorporate the ReLU activation functions for feature extraction, the third layer serves as a linear summarization and output module, and the fourth layer houses a softmax classifier for final classification.

#### 2.5.2. Solution to Bearing Defect Detection

The neural network presented in this paper exhibited similarities to the WDCNN model described in the literature [11], albeit with several implemented optimizations. Specifically, we removed the last convolution layer and pooling layer, adjusted the parameter settings of the convolution layer, replaced AdaBN with BN layers, and adopted alpha dropout for the dropout layers. These modifications were made to prioritize learning efficiency within our machine learning algorithm, ensuring a higher rate of model updates through the adoption of fewer layers. Moreover, as each set of data was trained separately during online learning, excessive connections between the training processes were unnecessary; thus, it was inappropriate to employ AdaBN, which was originally utilized in transfer learning.

The WDCNN architecture is designed to maximize information extraction by utilizing wide convolution kernels in the shallow layers while analyzing deep semantic features with small convolution kernels in the deeper layers. Multilayer narrow convolutions effectively segmentize fault features. The structure of the WDCNN in this paper comprised a first convolutional layer with 16 channels and a 64 × 1 convolution kernel followed by a second convolutional layer with 32 channels and a 3 × 1 convolution kernel; then, a third convolutional layer with 64 channels and a 3 × 1 convolution kernel; and, finally, a fourth convolutional layer with 16 channels and a 3 × 1 convolution kernel. Each of these layers was subsequently followed by a one-dimensional max-pooling layer of the size 2 × 1. The stride for the first convolutional layer was set to be 16, while, for the rest it, it was set to be 1. Additionally, each layer incorporated an activation function as well as batch normalization (BN). Specifically, the ReLU activation function was employed in the convolutional layers for linear correction. The formula of ReLU is as follows:

(12) $$ReLU\left(x\right)=\left\{\begin{array}{c} x,x>0\\ 0,x\leq 0 \end{array}\right.$$

The calculation formulas of the BN layer are as follows:

(13) $$\mu =\frac{1}{m}\sum_{i=1}^{m}x_{i}$$

(14) $$\sigma =\frac{1}{m}\sum_{i=1}^{m}{\left(x_{i}-\mu \right)}^{2}$$

(15) $$\hat{x_{i}}=\frac{x_{i}-\mu }{\sqrt{\sigma^{2}+\varepsilon }}$$

(16) $$y_{i}=\gamma \hat{x_{i}}+\beta$$

(17) $$f\left(x\right)=\frac{\gamma \omega }{\sqrt{\sigma^{2}+\varepsilon }}x+\frac{\gamma }{\sqrt{\sigma^{2}+\varepsilon }}\left(b-\mu \right)+\beta$$

Among these formulas, μ and σ represent the mean value and variance of each feature map, $\hat{x_{i}}$ represents the normalized data, and $y_{i}$ can be regarded as the output obtained through translation and scaling using β and γ. β and γ serve as shift factors and scale factors, respectively, both of which are learnable parameters that cannot be fixed but may vary with each training batch. By utilizing these parameters, the BN layer could effectively mitigate the internal covariate shift, thereby maintaining relative stability within a limited range for the overall parameter values. Additionally, the BN layer recalibrated its other parameters to ensure controllability while enhancing network stability for optimal training outcomes.

The alpha dropout layer was employed following the fully connected layer to effectively address overfitting issues during training and to maintain self-normalization. The specific principle involved applying Bernoulli distribution to selectively zero out certain elements, while the remaining elements were randomly adjusted in terms of scaling and shifting during each forward call to maintain a unit standard deviation. This approach ensured the constancy of both the mean and variance while activating negative saturation values in a random manner to self-regularize the data. To ensure an output with a unit standard deviation, alpha dropout was always paired with the SeLU activation function, which can be defined by the following formula:

(18) $$SeLU(x)=\left\{\begin{array}{c} \lambda \alpha \left(e^{x}-1\right),x<0\\ \lambda x,x\geq 0 \end{array}\right.$$

The values of λ and α in the formula were predetermined. SeLU ensured faster internal normalization compared to external normalization, leading to accelerated network convergence.

**Figure 5:**
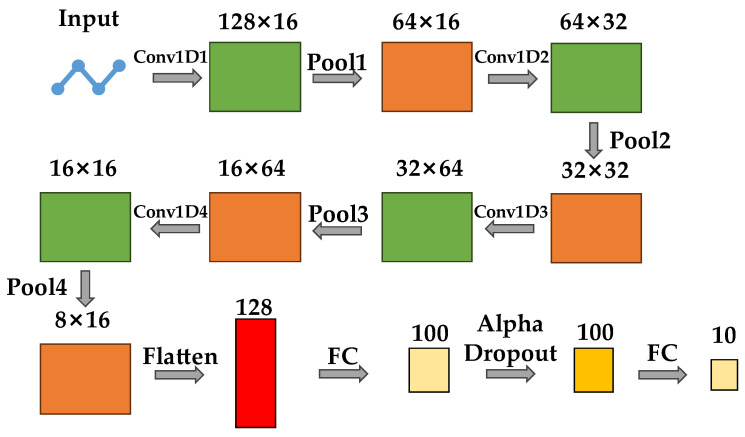
The structure of modified WDCNN in this paper.

The structure of the convolutional neural network presented in this paper is shown in Figure 5.

In Figure 5, the numerical value in each step represents the output size of the corresponding layer. The input denotes a one-dimensional signal from the CWRU dataset, while “Conv1d” refers to one-dimensional convolution. One-dimensional convolution involved performing a sliding-window operation on the one-dimensional input and utilized the input data within the convolutional window to compute an output value. As it operated only along the width of the data, its result was a two-dimensional sequence. For instance, 128 in “128 × 16” in Figure 5 represents the width of the output, while 16 indicates its depth or the number of channels. “Pool” refers to max pooling; “flatten” denotes the flattening operation, which transformed the two-dimensional data into a one-dimensional form; “FC” denotes the full connection layer; and “Alpha Dropout” refers to the dropout layer.

## 3. Experiments and Results

### 3.1. Experimental Environment

The hardware utilized in this paper employed a computer equipped with a 13th Gen Intel(R) Core(TM) i7-13700HX CPU, 16.00 GB of RAM, and an NVIDIA GeForce RTX 4060 graphics card with 8G of memory. The software environment was the Windows 11 operating system, which runs on the python 3.9 programming environment and mainly utilizes pytorch and the deep river framework while invoking library functions such as math, matplotlib, and others.

### 3.2. Data Preprocessing

#### 3.2.1. Data Preprocessing Method for Car Evaluation

Due to the limited amount of data in the dataset, the features extracted during training may have been insufficient. Therefore, employing data augmentation techniques was considered necessary. Data augmentation could enhance model generalization, mitigate overfitting, improve stability, and enable better adaptability to diverse scenarios and changes, thereby enhancing the overall model performance and effectiveness.

For the car evaluation dataset, which contains labeled text–numeric mixed data, category balancing was employed as a means of enhancing the data by increasing the number of samples categorized as “acceptable”, “good”, and “very good”, respectively, to match that categorized as “unacceptable”. This adjustment was made because there were 1210 instances labeled as “unacceptable”, which was the highest amount among all the categories. To achieve this balance, we first calculated the required number of enhanced samples for each category and then resampled from within each category using the sampling back technique to generate additional samples. Finally, these augmented samples were added to create an enhanced dataset consisting of a total of 4840 pieces of data.

The label features in the car evaluation dataset are easily identifiable, thus the one-hot conversion method was considered for data preprocessing, involving transforming the data into the approximate binary format using the pandas function.

#### 3.2.2. Data Preprocessing Method for Bearing Fault Detection

Similar to the case with the car evaluation dataset, due to the relatively small size of the training sample, overfitting could easily occur. Moreover, the dataset consists of one-dimensional fault diagnosis signals that exhibit unique timing and periodicity characteristics. Therefore, we devised a dedicated function to realize the random sampling of the data and to employ one of three techniques randomly: scaling, shifting, or adding noise to the sampled data to realize data augmentation. Among them, the shifting method refers to the overlapping sampling method mentioned in the literature [11] to ensure the maximum retention of the data’s original information and characteristics. The ratio between the augmented and original data was maintained at 1:1. Following data augmentation, there were 20,000 pieces of data. Prior to their input, normalization using river’s MinMaxScaler preprocessor was applied.

### 3.3. Experimental Processing and Data Comparison

#### 3.3.1. Experimental Results of Car Evaluation

The architecture of the neural network and its corresponding parameters for automobile quality inspection are presented in Table 3.

**Figure 6:**
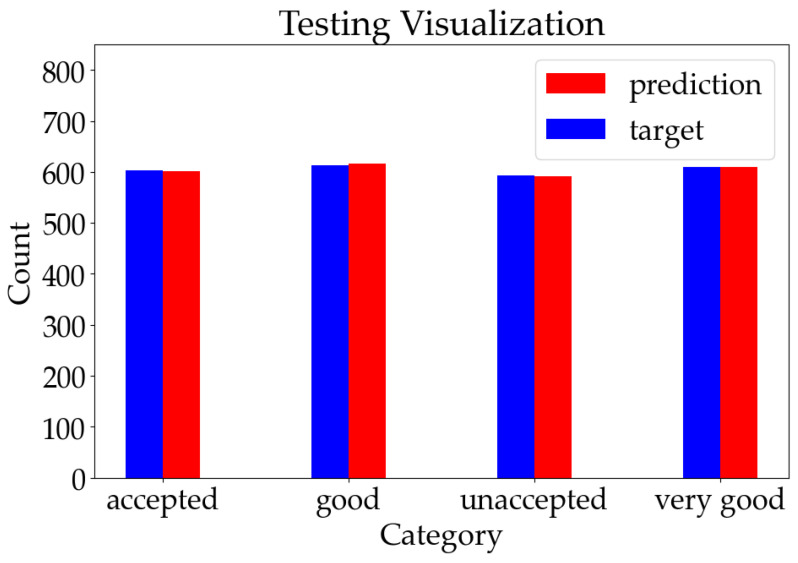
The test classification results for car evaluation.

Considering the simplicity of our network structure and the limited dataset size, we chose a batch size of one to ensure that each data point could undergo iteration during training. For evaluation purposes, half of our available data were allocated for training, while the remaining half was reserved for testing. In the FTRL optimizer, l1 regularization was set to 0.001, l2 regularization was set to 0.1, the alpha value was set to 0.1, and the beta value was set to 1. The specific reason for this is that, among these four hyperparameters, the alpha value regulates the update amplitude of each step during optimization iterations within a typical range of values between 0.1 and 1. A higher alpha value will lead to sparser feature weights; therefore, a conservative choice of setting it to a lower bound, such as 0.1, ensures better control over the training process. The beta value governs model sparsity and typically ranges from 0.1 to 1; however, it is commonly set to 1 to maintain an appropriate early learning rate without excessively hindering the training progress. L1 and l2 are utilized for regularization control, with parameter values usually selected in the range of 0.0001 to 1. Based on the insights gleaned from the literature sources [38] combined with the extensive experimentation conducted on our own datasets, we empirically determined that l1 should be set to 0.001, while l2 should be set to 0.1. With the appropriate values assigned to the learning rate and various parameters of FTRL, the accuracy of the training process gradually and smoothly improved until it reached a stable state after a certain number of iterations. Since each training process involved only one set of data and was relatively independent, the accuracy and loss value of the training process do not have substantial significance for the final classification results. They can only be maintained as records in the actual production process to facilitate subsequent inspection and sorting. The final test classification results are shown in Figure 6.

**Figure 7:**
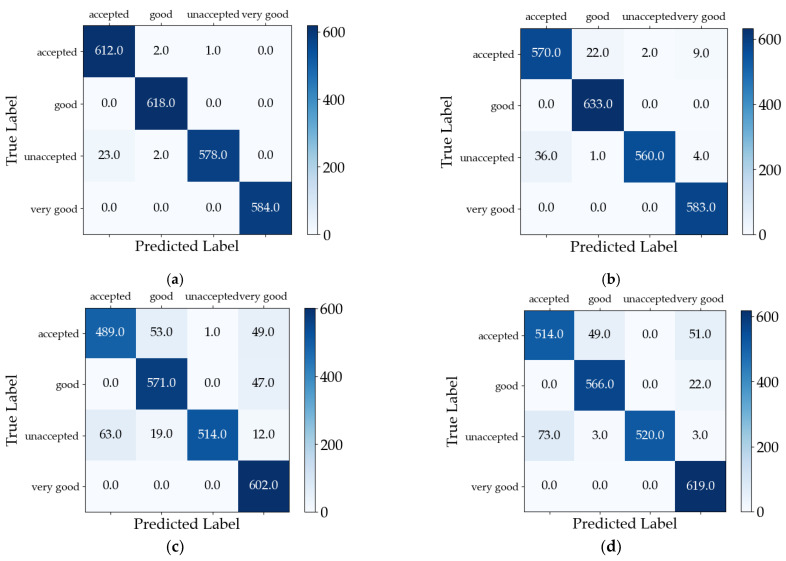
Comparison of confusion matrices for several methods tested on the car evaluation dataset: (**a**) online learning (ours), (**b**) support vector, (**c**) logistic regression, and (**d**) random forest.

The traditional machine learning methods provided in the original dataset and our online machine learning method were evaluated separately to better emphasize the advantages of online machine learning. The comparison of the classification results across all the methods is illustrated in Figure 7 in the format of confusion matrices.

Our online learning method achieved a classification accuracy rate of 98.843% as shown in Figure 6 and Figure 7a, accurately classifying 2392 out of the total 2420 test data. The classification accuracy rates of the other methods, obtained from the test and Figure 7, are presented in Table 4.

The advantages of online machine learning can still be observed to a certain extent when compared with other traditional offline learning methods, as demonstrated by the findings presented in Table 4, despite the disparities in the dataset processing and training approaches.

In order to further evaluate the performance of categorization after data augmentation, we conducted comparative experiments on both the augmented data and the original dataset under identical environmental conditions. Although the data enhancement ratio was 1:1, the method employed for enhancing the car evaluation dataset was category balancing, resulting in an inconsistent amount of data in every class undergoing enhancement. Also, it could not be ensured that the selected test set in the five-fold crossover test matched the original test set prior to enhancement. Therefore, the original dataset and enhanced dataset were directly compared through the training and testing of online machine learning using identical parameters. The proportion of test sets was 50%. The comparative results of the five tests are shown in Table 5.

The results presented in Table 5 demonstrate a marginal decrease in the classification accuracy following data augmentation. However, it is noteworthy that the mean difference in the classification accuracy across the five tests was only 0.314%. Additionally, the standard deviation of the test set before and after enhancement was found to be 0.160% and 0.088%, respectively, indicating that our classification model exhibited a level of stability even when exposed to perturbed input data.

#### 3.3.2. Experimental Results of Bearing Fault Detection

Table 6 systematically presents an overview of the specific details pertaining to our proposed WDCNN model.

**Figure 8:**
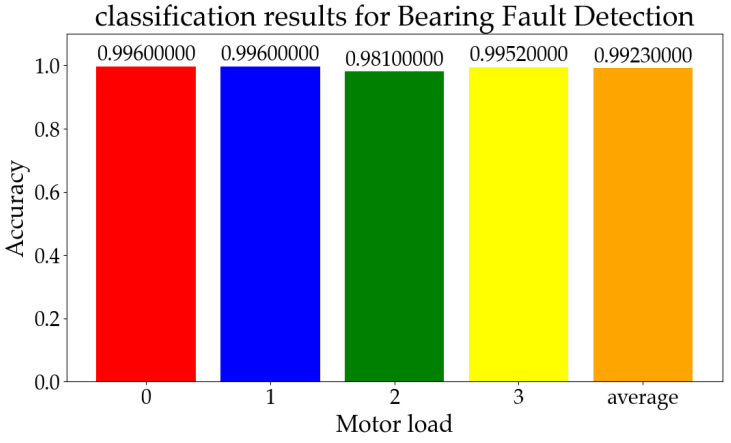
The test classification results for bearing fault detection.

The batch size and FTRL optimizer parameters remained consistent with those in Section 3.3.1, while the training sample proportion was adjusted to 50%. The fault types in the CWRU test set were categorized into four distinct levels based on horsepower, namely, levels 0, 1, 2, and 3. Consequently, it was imperative to conduct separate classified tests for each level. The final classification results based on horsepower are shown in Figure 8.

**Figure 9:**
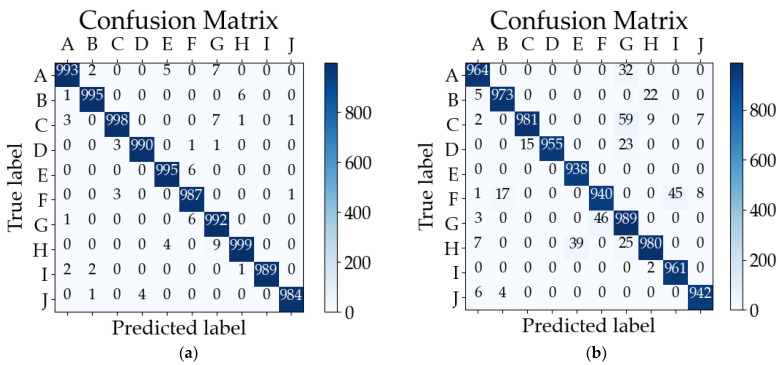

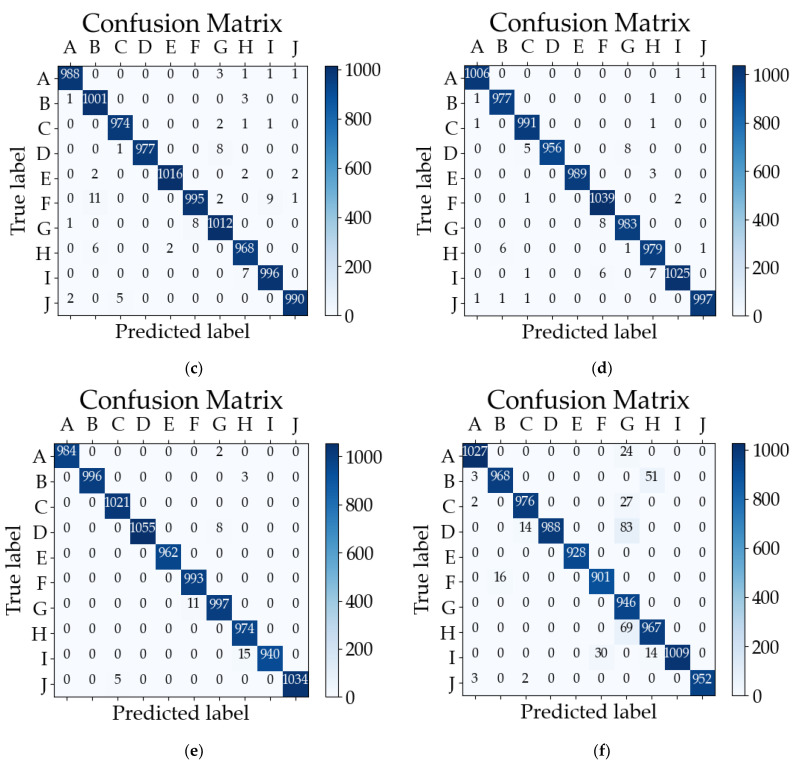
Comparison of confusion matrices for several methods tested on the CWRU dataset: (**a**) online learning (ours), (**b**) WDCNN, (**c**) Resnet, (**d**) AlexNet, (**e**) Lite CNN, and (**f**) VGG-16.

We validated multiple techniques by utilizing the structure and parameters presented in the partial references, which were tested on the CWRU dataset, resulting in the experimental data. The theoretical results claimed in the introduction of the original paper were supported by our tests, demonstrating a recognition accuracy comparable to those reported in the literature. The classification results obtained from testing these methods on the CWRU dataset are depicted in Figure 9 using confusion matrices. The correspondence between the category labels and letters can be found in Table 2.

The average classification accuracy rates, as obtained from the test results and Figure 9, are presented in Table 7.

The results demonstrate that our online learning approach achieved an accuracy comparable to those of the other deep learning methods, with the potential to attain near-perfect performance. This further validates the applicability of online machine learning models in real-world production scenarios.

The performance evaluation of the improved WDCNN was also conducted through five experiments, similar to Section 3.3.1, by comparing the processed CWRU dataset with the original dataset. However, it should be noted that the data enhancement method employed by the CWRU dataset involved generating random disturbances based on the original one-dimensional signal, thereby establishing correspondence between the enhanced and original data instances. In addition, the amount of data was the same for each category after data enhancement. Consequently, we could adopt a five-fold crossover experiment approach and ensure one-to-one correspondence between each datum in the divided test set before and after enhancement. The comparison results are presented in Table 8.

The results presented in Table 8 demonstrate that the enhanced dataset exhibited a difference of only 0.140% in its average classification accuracy compared to the original dataset; the standard deviation of the five-fold crossover experiment before and after enhancement was found to be 0.210% and 0.226%, respectively, thereby substantiating the improved WDCNN’s stability and resistance against interference.

## 4. Discussion

According to the data from the two experiments conducted in this paper for two distinct problems, it can be observed that the online machine learning method is more suitable for handling streaming data or time-series data. Additionally, better experimental results are achieved when there exists a certain correlation between the preceding and subsequent data instances. In the first experiment, which focuses on vehicle quality detection, a simplified neural network is employed in an online machine learning fashion. The model is trained using the car evaluation dataset by sequentially feeding 4840 pieces of data into the network. Although the final test accuracy falls slightly below those of the current highest record and some other offline algorithms, its training process exhibits a faster iteration speed due to the particularity of the small batch input and real-time model update. In the second experiment, targeting the bearing fault detection problem, a customized neural network based on the CWRU dataset is proposed for the online machine learning training process. The final test accuracy achieved with this approach compares favorably with those of other advanced offline learning algorithms while maintaining an efficient training rate. It highlights the advantage of online machine learning in handling strong temporal correlation information.

The experiment on bearing fault detection demonstrates that the online machine learning method achieves an identification accuracy comparable to that of the offline method as evidenced by a comparison with the literature [11]. Additionally, the online machine learning method exhibits a faster training speed and data iteration rate. This is attributed to the minimal impact of individual data inputs on the overall training process, enabling rapid and cost-effective learning steps. This enables the system to dynamically incorporate the latest received data, making online learning suitable for continuous streams of data. In scenarios with limited computing resources, online learning serves as an optimal solution since it discards the unnecessary data after incorporating new instances, resulting in significant space savings. Moreover, online learning algorithms are well suited for training systems on extensive datasets. These algorithms load and train on portions of the dataset sequentially until all the data have been utilized; however, this approach may incur higher training requirements. In summary, online machine learning boasts a wide range of applications and can outperform offline learning under specific conditions.

However, due to the inherent characteristics of online machine learning and some limitations in the experiments conducted in this paper, there are still certain shortcomings that need to be addressed. Firstly, the relatively high operation and maintenance costs must be resolved during the training process to ensure real-time sample stitching accuracy, to maintain real-time evaluation accuracy, and to roll back online models when necessary. Additionally, anomaly monitoring must be carried out to detect any performance declines quickly and to deal with them accordingly if bad data is used for training purposes. This incurs additional costs but mitigates risks effectively. Secondly, due to the limited experimental conditions and the small amount of data available for analysis in this study, some parameters may not have been optimally adjusted, resulting in suboptimal results being obtained. Furthermore, the model parameter updates may not necessarily trend towards optimal values during the training process given the unique framework of online machine learning algorithms themselves. Nonetheless, this paper demonstrates the efficacy of online machine learning algorithms and their advanced applications in industrial scenarios while substantiating the practical feasibility of employing this method for the quality inspection of industrial products through experimental validation.

## 5. Conclusions

The objective of this paper is to investigate the issue of quality inspection for industrial products in the manufacturing sector and to validate it using the car evaluation dataset and the CWRU dataset through online machine learning techniques. The experimental results demonstrate that, compared to other offline or deep learning methods, online learning achieves higher efficiency while maintaining superior recognition accuracy due to its reduced training iterations and unique model-updating mechanism. Additionally, this study also demonstrates that neural networks can implement online deep learning through Python’s deep river library. It should be noted that, due to various factors, such as data limitations and methodologies, the results obtained in this paper in terms of accuracy and loss values may not be optimal. However, this paper does prove that online machine learning can be utilized in real industrial activities and can complete basic detection and classification tasks under the condition of limited computing resources and high real-time requirements.

## Figures and Tables

**Table 1:** The different states of the automobile evaluation results and each indicator.

Label	States
Buying price	Very high, high, medium, low
Price of maintenance	Very high, high, medium, low
Number of doors	2, 3, 4, 5, and more
Capacity in terms of persons to be carried	2, 4, and more
The size of luggage boot	Small, medium, big
Estimated safety of the car	Low, medium, high
The results of car evaluation	Acceptable, unacceptable, good, very good

**Table 2:** The categories of dataset, the number of each type, and the letters representing each type.

Fault Type	Inner Raceway	Outer Raceway	Rolling Element	Normal
0 inch	0	0	0	1000
				J
0.007 inch	1000	1000	1000	0
	A	B	C	
0.014 inch	1000	1000	1000	0
	D	E	F	
0.021 inch	1000	1000	1000	0
	G	H	I	

**Table 3:** Neural networks for automotive quality classification.

Layer (Type)	Activation	In_Features	Out_Features
Linear1	ReLU	21	50
Linear2	ReLU	50	50
Linear3	ReLU	50	4
Linear4	softmax	4	4

**Table 4:** Test results compared to other methods validated on car evaluation dataset.

Methods	Mean Accuracy (%)
Support Vector Classification	96.942
Random Forest Classification	91.694
Logistic Regression	89.917
Online Learning (ours)	98.843

**Table 5:** Comparison of test results between enhanced car evaluation dataset and original dataset.

Dataset	Classification Accuracy (%)					Mean (%)	Standard Deviation (%)
Original dataset	98.719	98.760	98.554	99.050	98.802	98.777	0.160
Enhanced dataset	98.512	98.306	98.512	98.554	98.430	98.463	0.088

**Table 6:** Modified WDCNN model for bearing fault analysis.

Layer (Type)	Activation	Size of Filter and Pooling	Number of Channels
Conv1 (Conv1D)	ReLU	64 × 1	16
batch_normalization (BatchNormalization)			
Pool1 (MaxPooling1D)		2 × 1	
Conv2 (Conv1D)	ReLU	3 × 1	32
batch_normalization (BatchNormalization)			
Pool2 (MaxPooling1D)		2 × 1	
Conv3 (Conv1D)	ReLU	3 × 1	64
batch_normalization (BatchNormalization)			
Pool3 (MaxPooling1D)		2 × 1	
Conv4 (Conv1D)	ReLU	3 × 1	16
batch_normalization (BatchNormalization)			
Pool4 (MaxPooling1D)		2 × 1	
Fla1 (Flatten)			
FC1 (Fully Connected)	SeLU		
alpha_dropout (AlphaDropout)			
FC2 (Fully Connected)	softmax		

**Table 7:** Test results compared to other methods validated on CWRU dataset.

Methods	Mean Accuracy (%)
WDCNN [11]	96.230
Lite CNN [12]	99.560
Resnet [13]	99.170
AlexNet [14]	99.420
VGG-16 [15]	96.620
Online Learning (ours)	99.230

**Table 8:** Comparison of test results of enhanced CWRU dataset and original dataset.

Dataset	Classification Accuracy (%)					Mean (%)	Standard Deviation (%)
Original dataset	99.290	99.560	99.250	99.030	98.970	99.220	0.210
Enhanced dataset	99.180	99.420	99.140	98.850	98.810	99.080	0.226

## Data Availability

The car evaluation dataset’s source is http://archive.ics.uci.edu/dataset/19/car+evaluation (accessed on 20 October 2022). The CWRU dataset’s source is https://engineering.case.edu/bearingdatacenter/download-data-file (accessed on 1 November 2022).
